# Gene and Protein Accumulation Changes Evoked in Porcine Aorta in Response to Feeding with Two Various Fructan Sources

**DOI:** 10.3390/ani12223147

**Published:** 2022-11-14

**Authors:** Marta Marynowska, Agnieszka Herosimczyk, Adam Lepczyński, Marcin Barszcz, Adrianna Konopka, Aleksandra Dunisławska, Małgorzata Ożgo

**Affiliations:** 1 Department of Physiology, Cytobiology and Proteomics, Faculty of Biotechnology and Animal Husbandry, West Pomeranian University of Technology Szczecin, Klemensa Janickiego 29, 71-270 Szczecin, Poland; 2 Department of Animal Nutrition, The Kielanowski Institute of Animal Physiology and Nutrition, Polish Academy of Sciences, Instytucka 3, 05-110 Jabłonna, Poland; 3 Department of Animal Biotechnology and Genetics, Faculty of Animal Breeding and Biology, Bydgoszcz University of Science and Technology, Mazowiecka 28, 85-084 Bydgoszcz, Poland

**Keywords:** aorta, proteome, pig, 2D electrophoresis, mass spectrometry, inulin

## Abstract

**Simple Summary:**

Inulin-type fructans (ITFs), one of the best studied and most well-established prebiotic carbohydrates, have been shown to exert many health benefits to the host, including improvement in cardiovascular function. Various mechanisms are known to be involved in these processes, such as reduction of blood lipid levels by altering hepatic de novo lipogenesis, enhanced lipoprotein lipase activity in muscle, increased intestinal short-chain fatty acid production, elevated fecal excretion of bile salts and cholesterol and increased production of nitric oxide, known as endothelium-derived relaxing factor. Therefore, our overall aim was to assess the impact of complementary feeding with 4% of dried chicory root or 2% of native inulin on the aortic protein and gene changes in healthy nursery pigs. Our data clearly indicate that dietary inulin-type fructans have the potential to influence several structural and physiological alterations that are reflected both in the mRNA and protein levels in porcine aorta.

**Abstract:**

In this study, two different ITFs sources were incorporated into a cereal-based diet to evaluate possible aortic protein and gene changes in nursery pigs. The animals were fed two different experimental diets from the 10th day of life, supplemented with either 4% of dried chicory root (CR) or with 2% of native inulin (IN). After a 40-day dietary intervention trial, pigs were sacrificed at day 50 and the aortas were harvested. Our data indicate that dietary ITFs have the potential to influence several structural and physiological changes that are reflected both in the mRNA and protein levels in porcine aorta. In contrast to our hypothesis, we could not show any beneficial effects of a CR diet on vascular functions. The direction of changes of several proteins and genes may indicate disrupted ECM turnover (COL6A1 and COL6A2, MMP2, TIMP3, EFEMP1), increased inflammation and lipid accumulation (FFAR2), as well as decreased activity of endothelial nitric oxide synthase (TXNDC5, ORM1). On the other hand, the IN diet may counteract a highly pro-oxidant environment through the endothelin–NO axis (CALR, TCP1, HSP8, PDIA3, RCN2), fibrinolytic activity (ANXA2), anti-atherogenic (CAVIN-1) and anti-calcification (LMNA) properties, thus contributing to the maintenance of vascular homeostasis.

## 1. Introduction

Dietary supplementation with fiber may modulate the composition of intestinal microflora and thus interact with the host’s physiology. This effect is especially ascribed to inulin-type fructans (ITFs), a group of water soluble, non-digestible and fermentable carbohydrates. ITFs, including inulin (IN) and oligofructose (OF), constitute a class of compounds that meet the criteria of prebiotics. These carbohydrates are basically composed of linear β-(2→1)-linked D-fructosyl units varying, however, in their degree of polymerization (DP) and the lack of glucose at the end of fructose chain in the case of OF [1]. Native chicory inulin contains chains of DP ranging from 2 to 60, with an average DP of 10. Oligofructose is a short-chain fructan containing 2–10 fructose residues [2]. In nature, ITFs are the principal storage carbohydrates in many edible plants, and chicory root (*Cichorium intybus* L.) is considered as their main source used for industrial production. The meal obtained from the roots of chicory cultivars grown in Poland contains about 63% of IN and approximately 2% of OF [3]. Dried chicory root (CR) is characterized by a high content of prebiotic compounds, therefore, research attempts have been made to explore the use of this additive as a potential supplement in the diet of pigs.

The aorta (main artery) is the largest and most important blood vessel in systemic circulation. Arteries perform a wide variety of functions that include the maintenance of proper wall tension, ensuring proper blood pressure that enables its circulation. Due to the high content of elastic fibers, the aorta has the ability to change the phase nature of blood flow into a continuous flow to further sections of the arterial/vascular tree [4]. It should be highlighted that the use of the pig as a model of the human cardiovascular (CV) system has increased significantly over the past years due to its anatomical and physiological similarities. In general, the circulatory system, structure and size of the heart and vessels are almost identical to humans [5]. The CV system development from birth to 4 months of age is analogous to the development of the same system in teenagers [6,7]. In addition, the blood supply to the porcine coronary arteries as a conduction system is dominant on the right and is similar to 90% of the human population [8].

As recently reviewed ITFs have been shown to exert many health benefits to the host, including improvement in CV function [1,9], various mechanisms are known to be involved in these processes, such as decreased blood lipid levels by altering hepatic de novo lipogenesis, enhanced lipoprotein lipase activity in muscle, increased intestinal short-chain fatty acids (SCFAs) production and growth stimulation of SCFA-producing bacteria, especially those of *Akkermansia* genus [10], elevated fecal excretion of bile salts and cholesterol and increased production of nitric oxide known as endothelium-derived relaxing factor [11,12]. Results of our previous studies performed on nursery pigs support the lipid-lowering effects of ITFs, particularly on reducing plasma total cholesterol, HDL cholesterol [13] and triglyceride (TG) levels [14], with concomitant liver protein accumulation changes involved in cholesterol and TG metabolism [15]. Moreover, it was shown that both IN [16] and CR [14] diets resulted in profoundly increased accumulation of two apolipoproteins, namely A-I (apoA-I) and E (apoE), that are functionally related to HDL cholesterol metabolism. ApoA-I is also an activator of the lecithin-cholesterol acyltransferase (LCAT), an enzyme involved in the esterification of free cholesterol, which enables its transport in the blood plasma and limits its accumulation and the formation of atherosclerotic lesions in the aorta [17]. Additionally, APOE gene deficiency might be essential to development of hyperlipidemia and the progression of aortic atherosclerosis via aberrant hepatic lipid metabolism [18]. Of interest, a recent study by Deng et al. [12] provided strong evidence that ITFs can affect not only the abundance of plasma lipoproteins, but also their composition and functions, as IN feeding for 10 days decreased both plasma levels of ceramides (Cers) and also VLDL- and LDL-associated Cers in the atherogenic mouse model. It should be pointed out that increased plasma concentration of Cers, which are known precursors of complex sphingolipids, is a strong predictor of cardiovascular events. Thus, changes in sphingolipid metabolism via down-regulation of hepatic gene encoding neutral SMase accompanied by a decreased plasma ceramide level offers a novel mechanism of ITFs action [12]. Nevertheless, research aimed at exploring the potential of prebiotics for improving CV health condition have been performed mainly on individuals with diagnosed metabolic disorders or on experimental rodent models of CV diseases. In the literature, there is a lack of the evidence for the effect of ITFs on CV function in healthy, growing animals. To address this knowledge gap, in the present study, two different ITFs sources were used to evaluate a possible aortic protein and gene changes in nursery pigs. These ITFs were native chicory IN and dried CR.

We hypothesized that feeding a diet enriched either with 2% IN or 4% CR, naturally occurring dietary ingredients that are believed to be a rich source of ITFs and antioxidant, anticancer and anti-inflammatory phytochemicals, could enhance vascular function by increasing the abundance of aortic proteins and accumulation of genes involved in the regulation of vascular permeability and tone, fibrinolysis, as well as those displaying antioxidant and endothelium-dependent vasodilator effects.

## 2. Materials and Methods

### 2.1. Animal and Sample Collection

All experimental procedures were approved by the Local Commission of Ethics for the Care and Use of Laboratory Animals (No. 13/2012 of 23.05.2012, West Pomeranian University of Technology, Szczecin, Poland).

The present study was conducted on a total of 24 castrated male piglets (PIC × Penarlan P76) divided into three feeding groups (*n* = 8). Animal housing conditions have been previously characterized by Lepczyński et al. [15]. The feeding trial started at the 10th day of a piglet’s life and lasted 40 days in total. Until weaning, the piglets were kept with their sows (one group consisted of four sows with their litters). Initially, the piglets were fed with mother’s colostrum and milk for 9 days. The solid feed was introduced in the piglet diets on the 10th day of life. Dietary treatments were as follows: cereal-based diet without the addition of ITFs (control group—C) or with 2% IN (Inulin Orafti^®^ GR, BENEO GmbH, Mannheim, Germany), or with 4% of dried CR (VITAMIX, Niemcz, Poland). Diets were formulated to contain equal contents of ITFs. Ingredient compositions of the control and experimental diets are listed in Table 1. After weaning (28 day of life), two piglets from each litter were selected and allocated according to a given diet among separate pens (2 pens/group, 4 animals/pen) with free access to feed and water. The individual pig was regarded as the experimental unit. On the 50th day of life, at approximately 18 kg of body weight, piglets were stunned by electric shock and exsanguinated. The collected ascending aorta samples were washed twice in saline solution (0.9% NaCl, 4 °C) and then once with 20 mM Krebs-HEPES buffer (NaCl, KCl, CaCl_2_, MgSO_4_, K_2_HPO_4_, NaHCO_3_, pH 7.4, 4 °C; Noxygene, Elzach, Germany). Following washing, the tissue samples were immediately frozen in liquid nitrogen and then stored at −80 °C until further analysis.

### 2.2. Protein Extract Preparation

Aortic tissue samples (100 mg each) were homogenized with steel beads using mechanical homogenizer (Tissue Lyser, Qiagen, Hilden, Germany) at a frequency of 20,000 Hz for 5 min. Then the samples were further pulverized in 1 mL of lysis buffer (7 M urea, 2 M thiourea, 4% *w*/*v*, 1% *w*/*v* DTT, 2% *v*/*v* Biolyte, 1% *v*/*v* protease inhibitor cocktail, 0.1% *v*/*v* nuclease) at a frequency of 22,000 Hz for 50 min. After removal of insoluble tissue debris by a 20 min centrifugation at 22,000 g and 0 °C, protein extracts were used for two-dimensional electrophoresis.

### 2.3. Two-Dimensional Electrophoresis (2-DE)

Modified Bradford assay (Bio-Rad Protein Assay, Bio-Rad, Hercules, CA, USA) was applied to quantify the total protein concentration of the aortic samples. 1100 µg of protein was dissolved in lysis buffer (7 M urea, 2 M thiourea, 4% *w/v* CHAPS, 1% *w/v* DTT, 0.2% *w/v* 3–10 ampholytes, 650 µL final volume) and loaded onto 24 cm (linear) Ready Strip™ IPG Strips (Bio-Rad, Hercules, CA, USA) with pH ranges of 3–10. The isoelectrofocusing (IEF) was performed by a rapid ramp to 5000 V until all the IPG strips reached a total 90,000 Vh. After IEF, the IPG strips were incubated for 15 min in an equilibration buffer (6 M urea, 0.5 M Tris–HCl, pH 6.8, 2% *w/v* SDS, 30% *w/v* glycerol) with 1% DTT followed by 20 min in the same buffer containing 2.5% iodoacetamide. Second dimension (SDS-PAGE) was conducted on 12% SDS polyacrylamide gels (20 × 25 cm) at 40 V for 3.5 h and then at 100 V for 17 h at 15 °C using a Protean Plus™ Dodeca Cell™ electrophoretic chamber (Bio-Rad, Hercules, CA, USA). After SDS-PAGE separation, the gels were stained with colloidal Coomassie Brilliant Blue G-250 according to the protocol of Pink et al. [19]

### 2.4. Image Analysis

The gels were scanned using a GS-800™ Calibrated Densitometer (Bio-Rad, Hercules, CA, USA) and spots were detected and matched with the aid of a PDQuest Analysis software version 8.0.1 Advanced (Bio-Rad, Hercules, CA, USA). Protein spots were detected and manually landmarked to the master gel (that displayed the highest number of spots) to improve matching quality before the automatic matching. Following this step, only the spots that were present on at least six gels were further processed. The area-based method was used as the parameter for spot quantification after local regression (LOESS) normalization. The degree of difference between protein groups was expressed as an average ratio. To measure the variability within the group, the coefficient of variation was calculated for each experimental replicates. For the statistical analysis of the differences in relative abundance of protein spots Student’s *t*-test was used as integrated in the PDQuest software. Significance of the differences was set at the level of *p* ≤ 0.05. The experimental molecular masses were assessed using Precision Plus Protein™ Kaleidoscope™ Standard for SDS-PAGE (Bio-Rad, Hercules, CA, USA).

### 2.5. MALDI—TOF Mass Spectrometry Analysis and Bioinformatic Data Analysis

Statistically altered spots were excised from the gels and washed with a buffer (25 mM NH_4_HCO_3_ in 5% *v/v* acetonitrile (ACN)), followed by two washes with a solution containing of 25 mM NH_4_HCO_3_ in 50% *v/v* ACN. Next, they were dehydrated using 100% of ACN and vacuum dried. Finally, 12.5 µg trypsin/mL in 25 mM NH_4_HCO_3_ (Promega, Madison, WI, USA) was added and incubated overnight at 37 °C. After digestion, the peptides were extracted with 100% ACN, combined with the matrix solution (5 mg/mL CHCA, 0.1% *v/v* TFA, 50% *v/v* ACN) and placed onto a MALDI-MSP AnchorChip™ 600/96 plate (Bruker Daltonics, Germany) in a final volume of 1 µL. Droplets were allowed to dry at room temperature. External calibration was performed using a Peptide Mass Standard II (mass range 700–3200 Da, Bruker Daltonics, Germany). A Microflex™ MALDI-TOF (matrix-assisted laser desorption/ionization time of flight) mass spectrometer (Bruker Daltonics, Germany) was operated in a positive ion reflector mode. The data were searched against the SWISS-PROT and NCBI mammal databases for protein identification with the aid of the MASCOT search engine. The following search criteria were included: trypsin as an enzyme, carbamidomethylation of cystein as a fixed modification, methionine oxidation as a variable modification, mass tolerance to 150 ppm and a maximum of one missed cleavage site. The identification was assigned as positive only if the MASCOT score was significant (*p* < 0.05) and sequence coverage was at least 20%.

### 2.6. STRING Analysis of Protein Networks

Gene Ontology (GO) enrichment analysis and protein–protein interactions (PPI) of significantly altered proteins and genes were performed using STRING v11.5 [20]. Due to lack of comprehensive data for *Sus scrofa,* the human (*Homo sapiens*) genome was set as the reference. The medium confidence score (0.400) and identification of significant results based on a Benjamini–Hochberg False Discovery Rate (FDR)-adjusted *p*-value ≤ 0.05 were used to select the network of PPI. Functional association was made from the STRING cluster enrichment tables based on the analyzed tissue characteristics.

### 2.7. RNA Isolation

Aortic fragments (*n* = 6 per group) were added to 1 mL of Trizol (MRC, Cincinnati, OH, USA) and homogenized (TissueRuptor, Qiagen GmbH, Hilden, Germany) to isolate RNA. Next, chloroform (200 μL) was added into each tube of homogenate. The mixture was shaken vigorously and centrifuged (12,000 rpm, 15 min). The upper aqueous phase containing isolated RNA was collected. Additional purification was done with a commercial kit (Universal RNA Purification Kit, EURx, Gdansk, Poland). RNA quality and quantity were checked by electrophoresis (2% agarose gel) and spectrophotometer NanoDrop2000 (Scientific Nanodrop Products, Wilmington, NC, USA). The RNA was stored at −20 °C to further analysis.

### 2.8. Gene Expression Analysis

Gene accumulation analysis was performed by quantitative reverse transcription PCR (RT-qPCR). cDNA was synthesized using Maxima First Strand cDNA Synthesis Kit for RT-qPCR (Thermo Scientific/Fermentas, Vilnius, Lithuania), following the manufacturer’s recommendations. The qPCR reaction mixture included Maxima SYBR Green qPCR Master Mix (Thermo Scientific/Fermentas, Vilnius, Lithuania), 1 μM of each primer and diluted cDNA (140 ng). The RT-qPCR reaction for each sample was conducted in two technical replicates. Primer sequences for reference genes (ACTB and RPL4) were derived from the literature, while primers for target genes were designed by using NCBI Primer BLAST tool based on NCBI sequences [21]. The primer sequences are presented in Table 2. Thermal cycling was performed in a LightCycler 480 instrument II (Roche Diagnostics, Basel, Switzerland). The program consisted of initial denaturation (95 °C, 20 min) followed by 40 cycles of amplification (15 s, 95 °C), annealing (20 s, 58°) and elongation (20 s, 72 °C). Relative gene expression analysis was conducted separately for each experimental group by the ΔΔCt method [22]. Geometric Ct (cycle threshold) values of reference genes were used in further analysis [23]. The Student’s *t*-test (*p* < 0.05) was used to reveal any statistically significant differences.

## 3. Results

### 3.1. Analysis of Aorta Proteome

**Figure 1:**
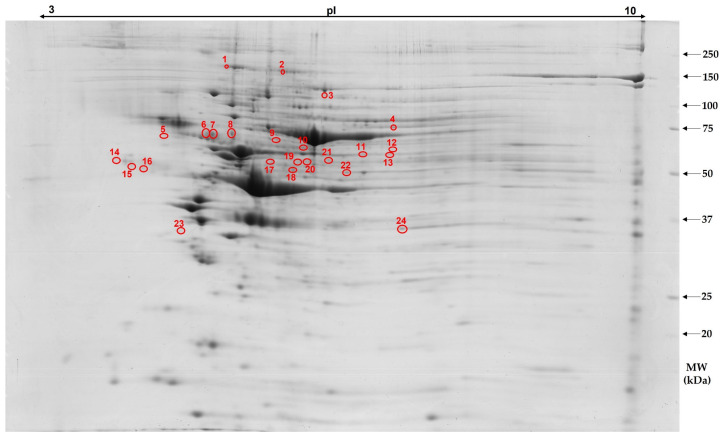
Representative two-dimensional electrophoresis gel image of porcine aorta proteome. Differentially expressed proteins between the animals fed standard diet (C) and experimental IN and CR diets are indicated by numbers. Protein spots numbers corresponds to those presented in the Table 3.

A comparative bioinformatic analysis of porcine aorta protein profiles showed 407–426 protein spots per each analyzed 2-D gel. The coefficient of variation was estimated at the level of 62.17%, 61.28% and 65.85% for the C, IN and CR group, respectively. A diet supplemented with 2% IN induced accumulation changes of 23 protein spots, of which 13 were successfully identified. Among these spots, eleven were down-regulated and two were up-regulated, compared to the control animals. In comparison with the control group, the CR diet significantly induced the accumulation of 31 protein spots, among which, 17 were successfully identified. Of these, fourteen were found to be down-accumulated, while three were up-accumulated. A representative 2-D gel image is shown in Figure 1, in which, all significantly changed proteins in response to both experimental diets are marked with the numbered circles. A list of aortic proteins and their average accumulation ratio along with mass spectrometric identification parameters is given in Table 3.

### 3.2. STRING Analysis of Protein Networks

**Figure 2:**
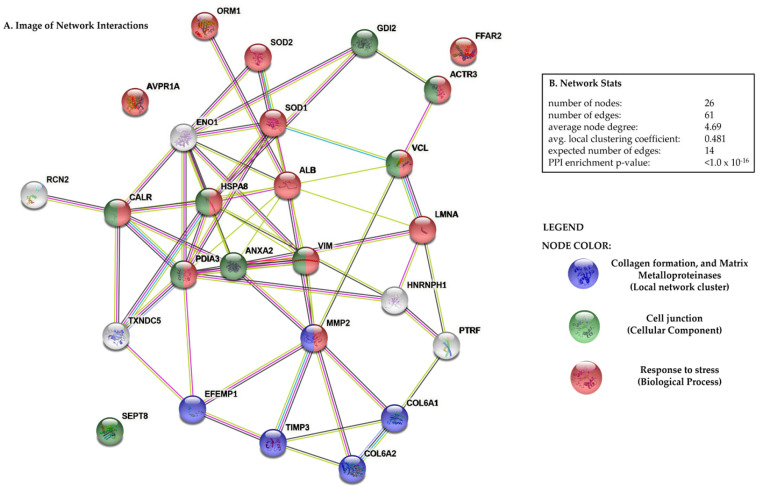
STRING analysis of differentially accumulated proteins and genes between the control and treatment groups in pig aorta. (**A**) Protein–protein interaction image displaying nodes and edges between all porcine aorta proteins and genes. Proteins and genes are represented as nodes. Various thickness of the edges indicates the strength and nature of the presented interactions detected between them. Different colors of nodes display selected GO terms such as biological process (response to stress), cellular component (cell junction) and local network cluster (STRING) (collagen formation, and matrix metalloproteinases) that are closely related with diet-induced changes in porcine aortic wall protein and gene expression patterns. Full name of proteins along with the corresponding genes can be found in Table 2 and Table 3. (**B**) Network statistics presents data concerning included number of nodes and edges, the average node degree, the average local clustering coefficient, the expected number of edges, and the protein–protein interaction (PPI) enrichment *p*-value.

Functional enrichment analysis was performed using the STRING software v. 11.5. Selecting as input the list of all protein spots and selected genes showing statistically significant alterations in response to the CR and IN diets, a GO enrichment analysis was performed from several aspects including biological process, cellular component and local network cluster (STRING). The protein–protein interactions (PPI) network analysis (Figure 2) revealed significant association between 26 proteins (*p*-value = 1.06 × 10^− 16^). This network was created using proteins (as nodes) and their known and predicted interactions (as edges). With different colors of nodes we have marked selected GO terms such as biological process (response to stress, *p* = 0.0254), cellular component (cell junction, *p* = 0.0392) and local network cluster (collagen formation, and matrix metaloproteinases, *p* = 0.0037) that are highly associated with diet-induced changes in porcine aortic wall protein and gene accumulation patterns.

### 3.3. Changes in the Gene Accumulation in Porcine Aorta

**Figure 3:**
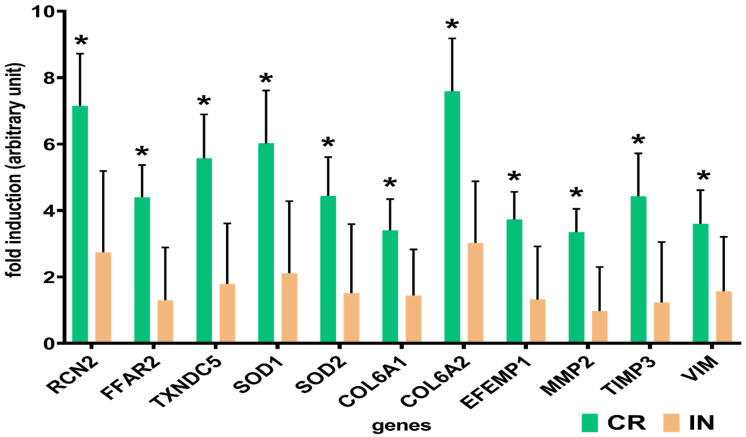
Differences in the relative accumulation of stress response, cytoskeletal and extracellular matrix-related genes in the aorta of pigs fed a diets enriched with 4% of dried chicory root (CR) or 2% of native chicory inulin (IN). Student’s *t*-test was carried out to determine the considerable differences between experimental and control groups. Values that differed significantly (*p* < 0.05) from that of the control group were marked with an asterisk. Fold induction indicates the average ratio of accumulation for genes found in the aorta of pigs fed a diet supplemented with 4% dried chicory root (CR) compared with the control (C) group and 2% of native inulin (IN) compared with the C group.

This study also investigated the impact of feeding various types of ITFs on changes in gene accumulation in the aortas of pigs. Quantitative PCR analysis was conducted on eleven genes: reticulocalbin 2 (RCN2), free fatty acid receptor 2 (FFAR2), thioredoxin domain-containing protein 5 (TXNDC5), superoxide dismutase 1 (SOD1), superoxide dismutase 2 (SOD2), collagen alpha—1 (VI) chain (COL6A1), collagen alpha—2 (VI) chain (COL6A2), EGF-containing fibulin-like extracellular matrix protein 1 (EFEMP1), matrix metallopeptidase 2 (MMP2), metallopeptidase inhibitor 3 (TIMP3), and vimentin (VIM). These genes are known to play vital functions in vascular biology related with stress response, structural integrity and extracellular matrix functions (Figure 3). These above-mentioned genes were all found to be significantly (*p* < 0.05) up-regulated in response to the CR diet when compared to the control group. Similar, but of lower strength, gene accumulation patterns were also observed in the group of pigs fed with the IN diet; however, the values did not reach significance.

Spot number corresponds to these in Figure 1. Protein and gene names show the unique identifier assigned by the UniProt/NCBI databases. This table also includes: average protein fold change, theoretical and experimental (estimated) isoelectric point (pI) and molecular weight (Mw) of each spot, and the percentage of the sequence coverage. Bold values denote statistical significance at the *p* ≤ 0.05 level. Functional annotation of proteins was based on UniProtKB database (http://www.uniprot.org, accessed on 13 August 2022)

Differentially accumulated proteins between the animals fed standard diet (C) and experimental IN and CR diets are indicated by numbers. Protein spots numbers corresponds to those presented in the Table 3.

## 4. Discussion

Here, we showed that a 40-day feeding with both ITFs diets has great potential to induce a wide variety of changes at either protein and mRNA level in the ascending aorta of nursery pigs. Given that the majority of genes and proteins perform their functions as a set of interactions we generated the PPI network to better understand the relationships that exist between differentially accumulated genes and proteins and to characterise which of them are involved in enriched GO terms, such as biological process (response to stress), cellular component (cell junction) and local network cluster (STRING) (collagen formation, and matrix metalloproteinases), that reflect vascular dynamic events.

### 4.1. Stress Response-Related Proteins

The most abundantly represented group of proteins in the PPI network were those involved in cellular stress response. The results of our study demonstrated that both experimental diets induced down-accumulation of CALR, TCP1, HSPA8 and PDIA3 proteins belonging to the group of molecular chaperones that are known to prevent protein aggregation and assist proper protein folding [25,26]. Due to the fact that altered accumulation of these proteins has a profound effects on many cellular functions, a clear relationship that may exist between these proteins in normal and diseased arteries still remains unclear. Recently, Hashikawa et al. [18] have shown that the induction of heat shock proteins (HSPs) promoted the progression of aortic atherosclerosis in ApoE knockout mice. Moreover, Pike et al. [27] demonstrated that NH_2_-terminal fragment of calreticulin (CALR) can inhibit endothelial cell proliferation in vitro and also suppress neovascularization in vivo. Additionally, a study by Androwiki et al. [28] suggested that enhanced protein disulfide isomerase (PDI) accumulation plays a crucial role in triggering oxidative stress and vascular dysfunction through increasing protein NOX1 expression and activity in conductance arteries. Taken together, our results may indicate that the decreased abundance of the aforementioned proteins in the porcine aorta may possibly counteract highly pro-oxidant environment arising from shear-induced signaling or/and stability of blood flow and thus contributing to the maintenance of vascular homeostasis. Accumulating evidence has revealed lowered levels of reactive oxygen species (ROS) and higher NO bioavailability in endothelial cells (EC) exposed to a regular blood flow and thus attenuating the local atherogenic hemodynamic environment [29]. Moreover, a decreased regulation of molecular chaperones was accompanied by a marked increase in mRNA accumulation level of both SOD-1 (6-fold) and SOD-2 (4-fold), encoding two isoforms of the antioxidant enzymes, in pigs fed the CR diet when compared with control ones. These results broadly supports our earlier observations, which showed that both ITFs diets have the potential to improve plasma, hepatic and kidney antioxidant status [13,30,31].

We also demonstrated that one of a member of the PDI family, thioredoxin domain containing protein 5 (TXNDC5), displayed nearly 6-fold increase in mRNA accumulation, whereas it showed an approximately 2-fold increase when measured at the protein level in the group of animals fed the CR diet. This finding was unexpected, especially given that this protein is frequently found overexpressed in many cardiovascular diseases, including myocardial fibrosis, as well as in the aortic atherosclerosis lesions [32,33]. Moreover, Cheng et al. [33] demonstrated that targeted deletion of TXNDC5 protects against endothelial dysfunction and aortic atherosclerosis as well as prevents down-regulation of endothelial nitric oxide synthase (eNOS) in ApoE knockout mice fed a high fat diet. Additionally, a decreased accumulation of alpha—1 acid glycoprotein (ORM1), a protein with documented vasodilatory effect, found in both experimental groups does not fully cover a putative health promoting effects on CV health. It is possible to hypothesize that some of the well-known beneficial effects of prebiotics on vascular endothelial function such as increased production of NO, a potent vasodilator and improved blood flow observed in animals with diagnosed metabolic disorders, are not revealed in healthy animals. Mechanism responsible for the aforementioned observation is still largely unknown and warrants further investigation.

### 4.2. Collagen Formation, and Matrix Metaloproteinases

Both composition and integrity of the extracellular matrix (ECM) are crucial elements of the physical characteristics of the aortic wall. The ECM in the vessels including aorta undergoes continuous physiological remodelling, through the constant process of proteolysis and renewed protein synthesis [34]. This stays in line with the results of our study where altered accumulation of several ECM proteins and genes encoding ECM-degrading enzymes, as well as physiological inhibitors of matrix metalloproteinases were found in response to both IN and CR diets. These include: collagen alpha—1 (VI) chain (COL6A1), collagen alpha—2 (VI) chain (COL6A2), EGF-containing fibulin-like extracellular matrix protein 1 isoform X1 (EFEMP1), matrix metalloproteinase 2 (MMP2) and tissue inhibitor of metalloproteinase 3 (TIMP3).

It should be pointed out that collagen fibers provide tensile strength to the aorta and limit distension at high blood pressure. Nevertheless, the appropriate ratio of elastin to collagen content in the aorta is essential for maintaining adequate mechanical properties. Our study revealed that feeding the CR diet triggered an increased accumulation of genes encoding COL6A1 and COL6A2, whereas an increase in protein levels was only observed in the case of COL6A1 in animals from the IN-fed group as compared to the control. Previous findings clearly indicate that COL6A1, in addition to the structural properties, also displays pro-angiogenic action, including endothelial cell proliferation, migration and survival, and its higher expression is associated with increased risk of hypertension and atherosclerosis [35,36]. A more recent study by Chen et al. [37] demonstrated that COL6A1 knockdown suppresses MMP2 expression in vascular smooth muscle cell (VSMC). Therefore, we may postulate that enhanced COL6A1 accumulation observed in our study might in turn cause a nearly four-fold increase in MMP2 gene expression in aortic tissue. As matrix metalloproteinases have an important function in regulating ECM remodelling and turnover, their activity needs to be tightly regulated by their endogenous inhibitors, known as tissue inhibitors of metalloproteinases (TIMPs) [38]. In the present study, elevated TIMP3 mRNA abundance in the CR group was evident, indicating physiological compensation to prevent a potentially harmful proteolytic environment in aortic tissue. Taken together, these results may suggest that ECM turnover might be disrupted in response to the CR diet but this needs to be investigated further.

Our study also demonstrated an increased EFEMP1 mRNA levels but it did not correlate with corresponding protein abundance, as this extracellular matrix-related glycoprotein was found to be down-expressed in response to the CR diet. This is probably due to the regulatory action of transcription factors that may modulate the rate of gene transcription and thus may result in decreased protein synthesis as in this case. It was previously evidenced by Lin et al. [39] that induced EFEMP1 accumulation may reduce MMP-2, MMP-9 and oxidative stress in the thoracic aorta in rats and thus improve vascular health and reduce cardiovascular risk factors for hypertension. Here, we observed an opposite effect as this protein was down-regulated with a concomitant increase in mRNA MMP2 abundance indicating potential adverse effects of the CR diet on vascular functions. Of note, significantly up-regulated accumulation of FFAR2 gene, that has been recently associated with inflammation [40] and lipid accumulation [41], was also found in this experimental group.

### 4.3. Cell Junction and Cytoskeletal Proteins

Both dietary ITFs sources resulted in different accumulation patterns of proteins in the porcine aorta associated with the cell junction and cytoskeleton, such as: vimentin (VIM), vinculin isoform X3 (VCL), actin related protein 3 (ACTR3), prelamin A/C (LMNA), septin 8 (SEPT 8), annexin A2 (ANXA2) and caveolae-associated protein 1 (CAVIN1). It is well established that the cytoskeleton is an important element of the vascular wall, ensuring its integrity and mechanical stability, and above all, it constitutes the first barrier against the shear stress of blood flow in the vascular system [42,43,44,45,46]. The CR diet was shown to up-regulate the VIM accumulation on both mRNA and protein levels in the ascending aorta of pigs. Vimentin, an intermediate filament protein, has been recently shown to modulate vasomotor tone through the endothelin–NO axis [47]. Moreover, VIM has been also implicated in the Notch signalling pathway that exists between endothelial cells (ECs) and vascular smooth muscle cells (VSMCs), suggesting a role in mechanotrasduction that regulates multilayer communication, as well as in structural homeostasis in response to shear stress [48]. Increased accumulation of VIM, observed in the current study, may be considered as a compensatory mechanism that maintains an optimal value of the shear stress, since knockout of VIM resulted in increased vascular tone, an impaired endothelium-dependent relaxation in rat carotid, and thus, leading to arterial stiffness [49]. This is further supported by a decreased accumulation of VCL, a protein responsible for the functional link of focal adhesions (FAs) to the actin cytoskeleton, found in the CR group. As previously reviewed, VIM provides resistance to mechanical stress through the regulation of both FAs and the actomyosin network [47]. Similar results were reported by Mott and Helmke [50] who observed the dynamic structural response of the actin, vimentin, paxillin and/or vinculin in aortic endothelial cells to onset of steady unidirectional shear stress. Moreover, we also showed that both sources of ITFs can ameliorate some of the toxic effects of prelamin A accumulation in vascular smooth muscle cells as both experimental diets had the potential to significantly down-regulate aortic LMNA protein. Emerging evidence has shown that this protein is involved in inducing persistent DNA damage signalling, thus, it has been shown to promote VSMCs calcification and aging [51].

Furthermore, we demonstrated lower accumulation of caveolae-associated protein 1 (CAVIN-1) in the aortic tissue of pigs fed the IN diet. As recently reviewed by He et al. [52], CAVIN-1 is a major component of caveolae, a subgroup of lipid rafts abundant in endothelial cells including vascular ECs that has been reported to play a critical function in orchestrating multiple cellular processes such as cholesterol homeostasis, nitric oxide production, and signal transduction. Recent evidence suggests that endothelial CAVIN-1 plays a pivotal role in the development and progression of atherosclerosis in mice [53]. This is further supported by the results of Wu et al. [54], who observed an increased accumulation of this protein in the arterial walls of hypercholesterolemic rabbits and ApoE knockout mice. This implies that decreased CAVIN-1 protein, found in our study, may reflect an anti-atherogenic effect of dietary native inulin.

A 40-day feeding with the IN diet also resulted in enhanced ANXA2 accumulation, a protein considered as endothelial cell co-receptor for plasminogen and tissue plasminogen activator (t-PA). Increased ANXA2 abundance mediates the increased plasmin generation on the endothelial lumina surface, thus showing fibrinolytic activity [55]. Our earlier proteomic study [16], performed on the same group of animals, showed that feeding a diet with 2% of inulin resulted in a decreased accumulation of plasminogen in the blood serum of pigs. These findings may suggest that the increased ANXA2 abundance reflects enhanced fibrinolysis that is of importance in maintaining the integrity of capillary beds.

Another interesting finding is that both experimental diets evoked down-accumulation of the reticulocalbin 2 (RCN2) protein, whereas a contradictory effect was observed at the gene level, as enhanced RCN2 mRNA abundance was noted, but only in the CR group. Divergence of gene and protein expression patterns can be due to regulatory action of transcription factors. Nevertheless, a recent study by Li et al. [56] showed that siRNA knockdown of RCN2 lowered basal blood pressure, blunted Ang II-induced hypertension and significantly enhanced the formation of NO by endothelial cells in mice.

## 5. Conclusions

In summary, our data clearly indicate that dietary ITFs have the potential to influence several structural and physiological changes that are reflected both in the mRNA and protein levels in porcine aorta. Nevertheless, in contrast to our hypothesis, we could not show evident beneficial effects of the CR diet on vascular functions. The direction of changes of several proteins and genes may indicate disrupted ECM turnover (COL6A1 and COL6A2, MMP2, TIMP3, EFEMP1), increased inflammation and lipid accumulation (FFAR2), as well as decreased activity of endothelial nitric oxide synthase (TXNDC5, ORM1). These unfavorable effects are rather surprising, particularly that such endothelial dysfunction, which is characterized by reduced NO bioavailability, excessive generation of reactive oxygen species and increased accumulation of inflammatory genes, has been implicated in the pathogenesis of hypertension and atherosclerosis. Additionally of interest is the fact that some CR diet-related changes in protein levels are not driven by corresponding changes in mRNA. However, based on the fact that the major limitation of our study was not determining the aortic wall histological changes, blood pressure and antioxidant activity measurements, it is not possible to fully assess the possible adverse effect of the CR diet on vascular function in pigs. Further specific studies are needed to elucidate it.

On the other hand, the results of our study showed some promising results in terms of improving vascular functions in the group of pigs fed a diet supplemented with 2% of native inulin. Based on the protein function and their pattern of changes, it may be suggested that the IN diet may counteract the highly pro-oxidant environment arising from shear-induced signaling or/and stability of blood flow through the endothelin–NO axis (CALR, TCP1, HSP8, PDIA3, RCN2), fibrinolytic activity (ANXA2), anti-atherogenic (CAVIN-1) and anti-calcification (LMNA) properties, thus contributing to the maintenance of vascular homeostasis.

Although these data still do not provide direct answers, we believe that they may lay a new foundation for future research and become an open field for discussion on the influence of ITF on the physiology of the blood vessels in growing animals.

## Figures and Tables

**Table 1:** Ingredient compositions of the control (C) and experimental diets (IN, CR).

Ingredient Composition %	C	IN	CR
Wheat	46.84	46.84	45.84
Barley	20	20	20
Corn starch	3.0	1.0	-
Full-fat soya bean	5.9	5.9	5.9
Whey	9.7	9.7	9.7
Fish meal	4.0	4.0	4.0
Spray-dried blood plasma	4.0	4.0	4.0
Soya bean oil	3.4	3.4	3.4
Calcium formate	0.3	0.3	0.3
Limestone	0.5	0.5	0.5
Dicalcium phosphate	0.6	0.6	0.6
Sodium chloride	0.07	0.07	0.07
L-lysine	0.61	0.61	0.61
DL-methionine	0.23	0.23	0.23
L-threonine	0.26	0.26	0.26
L-tryptophan	0.09	0.09	0.09
Mineral-vitamin premix ^1^	0.04	0.04	0.04
Aroma	0.01	0.01	0.01
Inulin ^2^	-	2	-
Dried chicory root ^3^	-	-	4

^1^ Premix composition (per kg): vitamin A 600,000 IU, vitamin D3 60,000 IU, vitamin E 3000 mg, vitamin K3 120 mg, vitamin B1 120 mg, vitamin B2 240 mg, vitamin B6 240 mg, nicotinic acid 1600 mg, pantothenic acid 800 mg, folic acid 160 mg, biotin 10 mg, vitamin B12 1.6 mg, choline chloride 12 g, Mg 0.8 g, Fe 6 g, Zn 5.6 g, Mn 2.4 g, Cu 6.4 g, I 40 mg, Se 16 mg, Co 16 mg. ^2^ Native chicory inulin with an average degree of polymerization of 10 (92% inulin, 8% other sugars: glucose, fructose, sucrose). ^3^ *Cichorium intybus* L. root (51.56% fructans). C—control group; IN—native chicory inulin group; CR—dried chicory root group.

**Table 2:** Primer sequences used in RT-qPCR reaction (F—Forward primer; R—Reverse primer).

Gene	Name	NCBI No.	Primer Sequence
RPL4 ^1^	ribosomal protein L4	100038029	F: CAAGAGTAACTACAACCTTC R: GAACTCTACGATGAATCTTC
ACTB ^1^	Beta—actin	414396	F: CACGCCATCCTGCGTCTGGA R: AGCACCGTGTTGGCGTAGAG
RCN2	reticulocalbin 2	100153840	F: AGATGCTGATGGCAGTCTTGA R: CAGTCATTCTGCAACACTCACC
FFAR2	free fatty acid receptor 2	100126285	F: GGACCCATCACAAGAAGCCA R: CTCTCCCCTCCAGCTCTGAT
TXNDC5	thioredoxin domain containing 5	100156354	F: CGTCCTCCATGCTGTTGTACT R: CTGTGCCCTCTCTGCATGTT
SOD1	superoxide dismutase 1	397036	F: CATTCCATCATTGGCCGCAC R: TGGGGACCTTTAGAAACCAGG
SOD2	superoxide dismutase 2	100154319	F: CTTGCAGATTGCCGCTTGTT R: CTCGTCTTCCTCACCTCACG
COL6A1	collagen alpha—1 (VI) chain	100623720	F: GCCTGGTCTACACCTCACG R: GCTCCTCAAAGGGACGACG
COL6A2	collagen type VI alpha—2 chain	100101552	F: CATCAGAAGTCCATGGCTGC R: TGAAGACTCTCAAGCAGCCA
EFEMP1	EGF containing fibulin extracellular matrix protein 1	100512046	F: TGGAGGCAGTTTGTAGAGGG R: GTGCTGGCAGATGATCAAGG
MMP2	matrix metallopeptidase 2	397391	F: AGTGTGTCCTTCAGCACGAA R: ATGCCATCCTAACGTGGCTG
TIMP3	TIMP metallopeptidase inhibitor 3	396775	F: CCACATCCTCATTGAGCTGC R: TTCATGCCAGCTTCTCTCCA
VIM	vimentin	100522394	F: TCAGTTTCACCCATGCGTCC R: TACGCACCAAAGCAAGTCAC

^1^ Nygard et al. [24].

**Table 3:** List of differentially accumulated protein spots found in the aorta of pigs fed experimental diets (IN, CR).

Spot No.	Accession No.	Protein Name	Gene Name	Ratio		SequenceCoverage %	MASCOT Score	Theoretical pI/Mw	Experimental pI/Mw
				IN/C	CR/C				
Stress response-related proteins									
5	NP_001167604	Calreticulin precursor	CALR	**0.51**	0.71	29	83	4.32/48.43	4.7/69.9
10	NP_001230356	T-complex protein 1 subunit alpha	TCP1	**0.45**	**0.52**	28	98	5.71/60.83	6.2/62.6
9	P19378	Heat shock cognate 71 kDa protein	HSPA8	**0.70**	**0.70**	25	71	5.24/70.99	5.9/67.1
21	NP_001182041	Protein disulfide—isomerase A3 precursor	PDIA3	0.70	**0.63**	34	108	5.93/57.28	6.5/56.2
12	Q4VIT4			0.55	**0.48**	23	64	6.23/57.14	7.2/59.9
11		Protein disulfide isomerase A3		1.29	**1.71**	20	62	6.23/57.14	6.9/58.9
17	XP_020955819	Thioredoxin domain-containing protein 5	TXNDC	2.58	**1.60**	33	135	5.94/48.70	5.9/54.3
15	AAA30983	Alpha—1 acid glycoprotein, partial	ORM1	**0.35**	**0.36**	36	81	5.83/21.11	4.3/53.0
16				**0.45**	**0.50**	36	94	5.83/21.11	4.5/51.8
6	P08835	Serum albumin	ALB	1.32	**0.60**	25	72	6.08/71.64	5.2/71.0
Cell junction and cytoskeletal proteins									
8	P02543	Vimentin	VIM	0.30	**1.30**	28	62	5.06/53.69	5.5/70.6
3	XP_005671131	Vinculin isoform X3	VCL	0.65	**0.72**	31	121	5.83/117.25	6.5/116.2
18	NP_001127815	Actin related protein 3	ACTR3	**0.52**	0.67	35	85	5.61/47.85	6.1/51.4
4	Q3ZD69	Prelamin A/C	LMNA	**0.64**	**0.78**	56	333	6.73/74.40	7.3/75.0
20	ANH21174	Septin 8	SEPT8	0.63	**0.57**	35	100	5.67/56.22	6.3/55.7
24	P19620	Annexin A2	ANXA2	**1.86**	1.27	57	200	6.49/38.76	7.3/35.5
23	Q6NZI2	Caveolae—associated protein 1	CAVIN1	**0.60**	0.57	17	62	5.51/43.45	4.8/35.0
Collagen formation, and matrix metalloproteinases									
1	XP_020926753	Collagen alpha—1 (VI) chain	COL6A1	**2.04**	1.34	12	79	5.23/109.69	5.4/192.6
2	XP_020938158	Collagen alpha—2 (VI) chain	COL6A2	**0.46**	1.06	24	123	6.13/110.44	6.0/169.9
7	XP_013851600	EGF-containing fibulin-like extracellular matrix protein 1 isoform X1	EFEMP1	0.58	**0.34**	29	81	5.16/52.30	5.3/70.4
Other proteins									
22	XP_020950937	Alpha—enolase isoform X1	ENO1	1.05	**0.71**	36	87	6.44/47.60	6.7/50.3
13	P31943	Heterogeneous nuclear ribonucleoprotein H	HNRNPH1	**0.39**	0.59	27	66	5.89/49.48	7.2/57.5
19	Q6Q7J2	Rab GDP dissociation inhibitor beta	GDI2	0.54	**0.49**	37	84	6.31/50.75	6.2/55.7
14	XP_020953684	Reticulocalbin—2 isoform X3	RCN2	**0.61**	**0.52**	35	87	4.18/36.21	4.1/57.3

Spot number indicates the number labelling of the spots in Figure 1. Protein and gene names are showed as in UniProt/NCBI database. The fold change, theoretical and experimental (estimated) isoelectric point (pI) and molecular weight (Mw) of each identified protein spot, and the percentage of the sequence coverage are also indicated. Differentially expressed spots at the level of *p* ≤ 0.05 are bolded. Proteins are grouped according to their function based on UniProtKB database (http://www.uniprot.org, accessed on 13 August 2022).

## Data Availability

Any data or material that support the findings of this study can be made available by the corresponding author upon request.

## References

[1] Hughes R.L., Alvarado D.A., Swanson K.S., Holscher H.D. (2022). The prebiotic potential of inulin-type fructans: A systematic review. Adv. Nutr..

[2] Bosscher D. (2009). Fructan prebiotics derived from inulin. Prebiotics and Probiotics Science and Technology.

[3] Gałązka I. (2002). The composition of chicory flour of selected chicory cultivars Polanowicka and Fredonia in relations to root sizes and date of harvest. Food Sci. Technol. Qual..

[4] Boudoulas K.D., Vlachopoulos C., Raman S.V., Sparks E.A., Triposciadis F., Stefanadis C., Boudoulas H. (2012). Aortic function: From the research laboratory to the clinic. Cardiology.

[5] Tsang H.G., Rashdan N.A., Whitelaw C.B.A., Corcoran B.M., Summers K.M., MacRae V.E. (2016). Large animal models of cardiovascular disease. Cell Biochem. Funct..

[6] Lelovas P.P., Kostomitsopoulos N.G., Xanthos T.T. (2014). A comparative anatomic and physiologic overview of the porcine heart. J. Am. Assoc. Lab. Anim. Sci..

[7] Swindle M.M., Smith A.C. (1998). Comparative anatomy and physiology of the pig. Scand. J. Lab. Anim. Sci..

[8] Swindle M.M., Makin A., Herron A.J., Clubb F.J., Frazier K.S. (2012). Swine as Models in biomedical research and toxicology testing. Vet. Pathol..

[9] Wu H., Chiou J. (2021). Potential benefits of probiotics and prebiotics for coronary heart disease and stroke. Nutrients.

[10] Catry E., Bindels L.B., Tailleux A., Lestavel S., Neyrinck A.M., Goossens J.F., Lobysheva I., Plovier H., Essaghir A., Demoulin J.B. (2018). Targeting the gut microbiota with inulin-type fructans: Preclinical demonstration of a novel approach in the management of endothelial dysfunction. Gut.

[11] Dos Reis S.A., da Conceição L.L., Rosa D.D., Dias M.M.d.S., Peluzio M.d.C.G. (2015). Mecanismos utilizados por los fructanos tipo inulina para mejorar el perfil lipídico. Nutr. Hosp..

[12] Deng P., Hoffman J.B., Petriello M.C., Wang C.Y., Li X.S., Kraemer M.P., Morris A.J., Hennig B. (2020). Dietary inulin decreases circulating ceramides by suppressing neutral sphingomyelinase expression and activity in mice. J. Lipid Res..

[13] Herosimczyk A., Lepczyński A., Ożgo M., Barszcz M., Jaszczuk-Kubiak E., Pierzchała M., Tuśnio A., Skomiał J. (2017). Hepatic proteome changes induced by dietary supplementation with two levels of native chicory inulin in young pigs. Livest. Sci..

[14] Lepczynski A., Herosimczyk A., Ozgo M., Skomial J., Taciak M., Barszcz M., Berezecka N. (2015). Dietary supplementation with dried chicory root triggers changes in the blood serum proteins engaged in the clotting process and the innate immune response in growing pigs. J. Physiol. Pharmacol..

[15] Lepczyński A., Herosimczyk A., Ożgo M., Marynowska M., Pawlikowska M., Barszcz M., Taciak M., Skomiał J. (2017). Dietary chicory root and chicory inulin trigger changes in energetic metabolism, stress prevention and cytoskeletal proteins in the liver of growing pigs—A proteomic study. J. Anim. Physiol. Anim. Nutr..

[16] Herosimczyk A., Lepczyński A., Ożgo M., Skomiał J., Dratwa-Chałupnik A., Tuśnio A., Taciak M., Barszcz M. (2015). Differentially expressed proteins in the blood serum of piglets in response to a diet supplemented with inulin. Pol. J. Vet. Sci..

[17] Boisvert W.A., Black A.S., Curtiss L.K. (1999). ApoA1 reduces free cholesterol accumulation in atherosclerotic lesions of ApoE-deficient mice transplanted with ApoE-expressing macrophages. Arterioscler. Thromb. Vasc. Biol..

[18] Hashikawa N., Ido M., Morita Y., Hashikawa-Hobara N. (2021). Effects from the induction of heat shock proteins in a murine model due to progression of aortic atherosclerosis. Sci. Rep..

[19] Pink M., Verma N., Rettenmeier A.W., Schmitz-Spanke S. (2010). CBB Staining protocol with higher sensitivity and mass spectrometric compatibility. Electrophoresis.

[20] Szklarczyk D., Gable A.L., Lyon D., Junge A., Wyder S., Huerta-Cepas J., Simonovic M., Doncheva N.T., Morris J.H., Bork P. (2019). STRING V11: Protein-protein association networks with increased coverage, supporting functional discovery in genome-wide experimental datasets. Nucleic Acids Res..

[21] Ye J., Coulouris G., Zaretskaya I., Cutcutache I., Rozen S., Madden T.L. (2012). Primer-BLAST: A tool to design target-specific primers for polymerase chain reaction. BMC Bioinform..

[22] Livak K.J., Schmittgen T.D. (2001). Analysis of relative gene expression data using real-time quantitative PCR and the 2^−ΔΔCT^ method. Methods.

[23] Vandesompele J., De Preter K., Pattyn F., Poppe B., Van Roy N., De Paepe A., Speleman F. (2002). Accurate normalization of real-time quantitative RT-PCR data by geometric averaging of multiple internal control genes. Genome Biol..

[24] Nygard A.-B., Jørgensen C.B., Cirera S., Fredholm M. (2007). Selection of reference genes for gene expression studies in pig tissues using SYBR green QPCR. BMC Mol. Biol..

[25] Biwer L.A., Askew-Page H.R., Hong K., Milstein J., Johnstone S.R., Macal E., Good M.E., Bagher P., Sonkusare S.K., Isakson B.E. (2020). Endothelial calreticulin deletion impairs endothelial function in aged mice. Am. J. Physiol. Heart Circ. Physiol..

[26] Chang Y.X., Lin Y.F., Chen C.L., Huang M.S., Hsiao M., Liang P.H. (2020). Chaperonin-containing TCP-1 promotes cancer chemoresistance and metastasis through the AKT-GSK3β-β-Catenin and xiap-survivin pathways. Cancers.

[27] Pike S.E., Yao L., Jones K.D., Cherney B., Appella E., Sakaguchi K., Nakhasi H., Teruya-Feldstein J., Wirth P., Gupta G. (1998). Vasostatin, a calreticulin fragment, inhibits angiogenesis and suppresses tumor growth. J. Exp. Med..

[28] Androwiki A.C.D., Camargo L.d.L., Sartoretto S., Couto G.K., Ribeiro I.M.R., Veríssimo-Filho S., Rossoni L.V., Lopes L.R. (2015). Protein disulfide isomerase expression increases in resistance arteries during hypertension development. Effects on Nox1 NADPH oxidase signaling. Front. Chem..

[29] Hsieh H.-J., Liu C.-A., Huang B., Tseng A.H., Wang D.L. (2014). Shear-induced endothelial mechanotransduction: The interplay between Reactive Oxygen Species (ROS) and Nitric Oxide (NO) and the pathophysiological implications. J. Biomed. Sci..

[30] Herosimczyk A., Lepczyński A., Ożgo M., Barszcz M., Marynowska M., Tuśnio A., Taciak M., Markulen A., Skomiał J. (2018). Proteome changes in ileal mucosa of young pigs resulting from different levels of native chicory inulin in the diet. J. Anim. Feed Sci..

[31] Lepczyński A., Herosimczyk A., Barszcz M., Ożgo M., Michałek K., Grabowska M., Tuśnio A., Szczerbińska D., Skomiał J. (2021). Diet supplemented either with dried chicory root or chicory inulin significantly influence kidney and liver mineral content and antioxidative capacity in growing pigs. Animal.

[32] Shih Y.-C., Chen C.-L., Zhang Y., Mellor R.L., Kanter E.M., Fang Y., Wang H.-C., Hung C.-T., Nong J.-Y., Chen H.-J. (2018). Endoplasmic reticulum protein TXNDC5 augments myocardial fibrosis by facilitating extracellular matrix protein folding and redox-sensitive cardiac fibroblast activation. Circ. Res..

[33] Cheng S.H., Yeh C.F., Fang Y., Yang K.C. (2018). Endoplasmic reticulum protein thioredoxin domain containing 5 (TXNDC5) is a novel mediator of endothelial dysfunction and atherosclerosis. Eur. Heart J..

[34] Jana S., Hu M., Shen M., Kassiri Z. (2019). Extracellular matrix, regional heterogeneity of the aorta, and aortic aneurysm. Exp. Mol. Med..

[35] Nandakumar P., Lee D., Richard M.A., Tekola-Ayele F., Tayo B.O., Ware E., Sung Y.J., Salako B., Ogunniyi A., Gu C.C. (2017). Rare coding variants associated with blood pressure variation in 15,914 individuals of african ancestry. J. Hypertens..

[36] Sleptsov A.A., Nazarenko M.S., Lebedev I.N., Skriabin N.A., Frolov A.V., Popov V.A., Barbarash L.S., Puzyrev V.P. (2014). Somatic genome variations in vascular tissues and peripheral blood leukocytes in patients with atherosclerosis. Genetika.

[37] Chen Z., Wu Q., Yan C., Du J. (2019). COL6A1 Knockdown suppresses cell proliferation and migration in human aortic vascular smooth muscle cells. Exp. Ther. Med..

[38] Moore L., Fan D., Basu R., Kandalam V., Kassiri Z. (2012). Tissue inhibitor of metalloproteinases (TIMPs) in heart failure. Heart Fail. Rev..

[39] Lin Z., Wang Z., Li G., Li B., Xie W., Xiang D. (2016). Fibulin-3 may improve vascular health through inhibition of MMP-2/9 and oxidative stress in spontaneously hypertensive rats. Mol. Med. Rep..

[40] Kim M.H., Kang S.G., Park J.H., Yanagisawa M., Kim C.H. (2013). Short-chain fatty acids activate GPR41 and GPR43 on intestinal epithelial cells to promote inflammatory responses in mice. Gastroenterology.

[41] Hong Y.-H., Nishimura Y., Hishikawa D., Tsuzuki H., Miyahara H., Gotoh C., Choi K.-C., Feng D.D., Chen C., Lee H.-G. (2005). Acetate and propionate short chain fatty acids stimulate adipogenesis via GPCR43. Endocrinology.

[42] Schiffers P.M.H., Henrion D., Boulanger C.M., Colucci-Guyon E., Langa-Vuves F., van Essen H., Fazzi G.E., Lévy B.I., de Mey J.G.R. (2000). Altered flow-induced arterial remodeling in vimentin-deficient mice. Arterioscler. Thromb. Vasc. Biol..

[43] Ivaska J., Vuoriluoto K., Huovinen T., Izawa I., Inagaki M., Parker P.J. (2005). PKCε-mediated phosphorylation of vimentin controls integrin recycling and motility. EMBO J..

[44] Nieminen M., Henttinen T., Merinen M., Marttila–Ichihara F., Eriksson J.E., Jalkanen S. (2006). Vimentin function in lymphocyte adhesion and transcellular migration. Nat. Cell Biol..

[45] Ivaska J., Pallari H.M., Nevo J., Eriksson J.E. (2007). Novel functions of vimentin in cell adhesion, migration, and signaling. Exp. Cell Res..

[46] Kwak H.I., Kang H., Dave J.M., Mendoza E.A., Su S.C., Maxwell S.A., Bayless K.J. (2012). Calpain-mediated vimentin cleavage occurs upstream of MT1-MMP membrane translocation to facilitate endothelial sprout initiation. Angiogenesis.

[47] Ridge K.M., Eriksson J.E., Pekny M., Goldman R.D. (2022). Roles of vimentin in health and disease. Genes Dev..

[48] Zhang J., Henrion D., Ebrahimian T., Benessiano J., Colucci-Guyon E., Langa F., Lévy B.I., Boulanger C.M. (2001). Increased contribution of l-arginine–nitric oxide pathway in aorta of mice lacking the gene for vimentin. J. Cardiovasc. Pharmacol..

[49] Langlois B., Belozertseva E., Parlakian A., Bourhim M., Gao-Li J., Blanc J., Tian L., Coletti D., Labat C., Ramdame-Cherif Z. (2017). Vimentin knockout results in increased expression of sub-endothelial basement membrane components and carotid stiffness in mice. Sci. Rep..

[50] Mott R.E., Helmke B.P. (2007). Mapping the dynamics of shear stress-induced structural changes in endothelial cells. Am. J. Physiol. Cell Physiol..

[51] Liu Y., Drozdov I., Shroff R., Beltran L.E., Shanahan C.M. (2013). Prelamin a accelerates vascular calcification via activation of the DNA damage response and senescence-associated secretory phenotype in vascular smooth muscle cells. Circ. Res..

[52] He J., Cui Z., Zhu Y. (2021). The role of caveolae in endothelial dysfunction. Med. Rev..

[53] Fernández-Hernando C., Yu J., Suárez Y., Rahner C., Dávalos A., Lasunción M.A., Sessa W.C. (2009). Genetic evidence supporting a critical role of endothelial caveolin-1 during the progression of atherosclerosis. Cell Metab..

[54] Wu C.-C., Wang S.-H., Kuan I.-I., Tseng W.-K., Chen M.-F., Wu J.-C., Chen Y.-L. (2009). OxLDL upregulates caveolin-1 expression in macrophages: Role for caveolin-1 in the adhesion of OxLDL-treated macrophages to endothelium. J. Cell. Biochem..

[55] He X., Drelich A., Yu S., Chang Q., Gong D., Zhou Y., Qu Y., Yuan Y., Su Z., Qiu Y. (2019). Exchange protein directly activated by CAMP plays a critical role in regulation of vascular fibrinolysis. Life Sci..

[56] Li J., Cechova S., Wang L., Isakson B.E., Le T.H., Shi W. (2019). Loss of reticulocalbin 2 lowers blood pressure and restrains ANG II-induced hypertension in vivo. Am. J. Physiol. Renal Physiol..

